# Inequalities at Age of Diagnosis of Severe Mental Illness in England

**DOI:** 10.1001/jamanetworkopen.2024.57371

**Published:** 2025-01-31

**Authors:** Sheena E. Ramsay, Ziyi Cai, David R. Sinclair, Matthew Prina, David P. J. Osborn, Ashley J. Adamson, Eileen Kaner, Emma A. Adams

**Affiliations:** 1 Population Health Sciences Institute, Newcastle University, Newcastle upon Tyne, United Kingdom; 2 Division of Psychiatry, University College London, London, United Kingdom; 3 Camden & Islington NHS Foundation Trust, London, United Kingdom

## Abstract

This cohort study evaluates whether socioeconomic factors, including sex, race and ethnicity, material deprivation, and rurality) are associated with the age at which patients are diagnosed with severe mental illness.

## Introduction

Severe mental illness (SMI) (schizophrenia, bipolar disorder, and other nonorganic psychotic illness) is associated with worse health outcomes.^1,2^ Similar to other chronic diseases, early intervention would yield better treatment response, prevent disease progression, and reduce lifetime disability.^3^ We investigated social inequalities (pertaining to socioeconomic deprivation, sex, race and ethnicity, and urban or rural residence) in age at recorded diagnosis of SMI in primary care.

## Methods

Following the STROBE reporting guideline, this cohort study used data from the Clinical Practice Research Datalink (CPRD) Aurum datasets containing anonymized routinely collected records for 19 million people registered in 738 general practices in England, broadly representative of primary care populations.^4^ Data were self-reported and included age, sex, race and ethnicity, and medical events (including diagnoses). Data were linked to the English Index of Multiple Deprivation, a composite index of area-level socioeconomic deprivation, urban-rural classification based on patient household address (97.7% of sample), and ethnicity records (97.9% of sample). The sample comprised individuals with first diagnosis of SMI from January 1, 2000, to September 31, 2022. SMI was defined as schizophrenia, bipolar disorder, or other nonorganic psychotic illness and validated primary care code lists were used for identifying diagnosis of SMI.^2^ We excluded patients with a registration period of less than 1 year, or date of first SMI diagnosis out of the study period, or date of first SMI diagnosis within first 12 months of registration to avoid including patients who recently moved practices and better ascertain first diagnosis of SMI. Ethical approval was obtained from the Independent Scientific Advisory Committee of the CPRD. Because the study included pseudonymized electronic health care records, CPRD determined informed consent was not required, and data were deidentified and approved.

Descriptive statistics of age at first diagnosis of SMI (median [IQR] values) were calculated. The proportion of people first diagnosed with SMI at specific age (age distribution at first diagnosis of SMI) and cumulative proportion were estimated. Linear regression models examined the associations between social factors (sex, race and ethnicity, urban or rural areas, and deprivation) and age at recorded diagnosis of SMI; 2-tailed *P* < .05 was considered statistically significant. Statistical analysis was performed from October 18 to November 21, 2023, using R software, version 4.3.1 (R Project for Statistical Computing).

## Results

Among 96 270 individuals who received a diagnosis of a SMI during the study period, the median age at diagnosis of SMI was 40 years (IQR, 28-57 years). The median age at diagnosis was lower in men (37 years) compared with women (44 years) as well as among Black, Asian, and mixed ethnic groups compared with White populations (Table). Compared with those in the least-deprived quintile (quintile 1), age at diagnosis of SMI for those in quintiles 4 and 5 (the most deprived quintiles) was −4.40 (95% CI, −4.83 to −3.97) years and −5.39 (95% CI, −5.81 to −4.98) years, respectively. Age at diagnosis of SMI was also lower among those residing in urban areas vs rural areas. These associations remained in adjusted regression models.

**Table.** Median Age at First Diagnosis of Severe Mental Illness and Estimates From Linear Regression According to Sex, Race and Ethnicity, Socioeconomic Deprivation, and Rural or Urban Residence Among the Study Population

Characteristic	No. (%) of individuals	Age, median (IQR), y	Linear regression			
			Unadjusted		Adjusted	
			β (95% CI)	*P* value	β (95% CI)	*P* value
Sex^a^						
Female	50 132 (52.1)	44 (30-62)	1 [Reference]	NA	1 [Reference]	NA
Male	46 135 (47.9)	37 (26-52)	−6.64 (−6.89 to −6.39)	<.001	−6.82 (−7.07 to −6.58)	<.001
Race and ethnicity						
Asian	7693 (8.0)	35 (25-47)	−7.42 (−7.88 to −6.96)	<.001	−6.81 (−7.28 to −6.35)	<.001
Black	7356 (7.6)	34 (24-47)	−7.77 (8.84 to −7.29)	<.001	−7.62 (−7.66 to −6.66)	<.001
Mixed	2045 (2.1)	29 (22-40)	−13.41 (−14.28 to −12.54)	<.001	−12.45 (−13.30 to −11.60)	<.001
White	76 895 (79.9)	42 (29-59)	1 [Reference]	NA	1 [Reference]	NA
Other^b^	255 (0.3)	34 (24-43)	−9.05 (−11.48 to −6.62)	<.001	−7.79 (−10.15 to −5.43)	<.001
Unknown	2026 (2.1)	42 (28-70)	2.81 (1.94 to 3.68)	<.001	1.73 (0.87 to 2.58)	<.001
Socioeconomic deprivation quintile^c^						
Quintile 1 (least deprived)	13 016 (13.5)	44 (29-64)	1 [Reference]	NA	1 [Reference]	NA
Quintile 2	14 685 (15.3)	42 (28-62)	−1.04 (−1.51 to −0.5)	<.001	−1.14 (−1.60 to −0.69)	<.001
Quintile 3	17 181 (17.9)	41 (28-58)	−2.57 (−3.03 to −2.12)	<.001	−2.16 (−2.60 to −1.71)	<.001
Quintile 4	22 618 (23.5)	39 (28-55)	−4.40 (−4.83 to −3.97)	<.001	−3.22 (−3.65 to −2.9)	<.001
Quintile 5 (most deprived)	28 770 (29.9)	39 (27-53)	−5.39 (−5.81 to −4.98)	<.001	−4.36 (−4.79 to −3.93)	<.001
Residence						
Rural	10 295 (10.7)	45 (30-65)	1 [Reference]	NA	1 [Reference]	NA
Urban	85 975 (89.3)	40 (28-56)	−4.54 (−4.95 to −4.13)	<.001	−2.45 (−2.86 to −2.03)	<.001

Abbreviation: NA, not applicable.

^a^ The totals given differ from the total in text because there were missing data for 3 participants.

^b^ This category was created by the Clinical Practice Research Datalink to reflect race and ethnicity recorded other than Asian, Black, mixed, or White.

^c^ Per the English Index of Multiple Deprivation.

## Discussion

To our knowledge, this study is the first in England to use primary care records to investigate socioeconomic inequalities in age at SMI diagnosis. Our findings highlight that populations with greater risk of SMI also receive diagnoses at earlier ages. Previous studies show inequalities in SMI prevalence and incidence according to deprivation and ethnicity.^5,6^

A study limitation is that the CPRD does not cover all general practices in England or patients moving to new practices. As an observational study, the findings cannot establish causality. Furthermore, the role of unmeasured confounders (eg, access to mental health services) cannot be ruled out.

Diagnosis at an earlier age provides opportunities for earlier treatment and preventing disease progression, particularly in groups with higher incidence of SMI. It is also indicates that these populations are disproportionately affected by factors leading to SMI. Further research should explore provision (and receipt) of timely interventions for SMI in different populations.

## Notes

Supplement.
Data Sharing Statement
