# Supplementary material for: Inequalities at Age of Diagnosis of Severe Mental Illness in England
Source: JAMA Netw Open. 2025 Jan 31;8(1):e2457371. doi: 10.1001/jamanetworkopen.2024.57371 (PMC11786225; doi:10.1001/jamanetworkopen.2024.57371)
Supplement: Supplement. — Data Sharing Statement [file jamanetwopen-e2457371-s001.pdf]

Data Sharing Statement

Ramsay. Inequalities at Age of Diagnosis of Severe Mental Illness in England. *JAMA Netw Open*. Published January 31, 2025. doi:10.1001/jamanetworkopen.2024.57371

Data
Data available: No

Additional Information
Explanation for why data not available: Data in the study are from the Clinical Practice Research Datalink (CPRD) data obtained under license from the UK Medicines and Healthcare products Regulatory Agency. CPRD contains patient-level data which are not publicly available to protect patient confidentiality. However, researchers can access the data by contact CPRD. Data are made available by CPRD to researchers with an approved research application. Details of the application process and conditions of access are available at https://www.cprd.com/data-access.
